# Diel, daily, and spatial variation of coral reef seawater microbial communities

**DOI:** 10.1371/journal.pone.0229442

**Published:** 2020-03-11

**Authors:** Laura Weber, Amy Apprill

**Affiliations:** 1 Marine Chemistry and Geochemistry Department, Woods Hole Oceanographic Institution, Woods Hole, MA, United States of America; 2 MIT-WHOI Joint PhD Program in Biological Oceanography, Woods Hole, MA, United States of America; Australian Bureau of Agricultural and Resource Economics and Sciences, AUSTRALIA

## Abstract

Reef organisms influence microorganisms within the surrounding seawater, yet the spatial and temporal dynamics of seawater microbial communities located in proximity to corals are rarely investigated. To better understand reef seawater microbial community dynamics over time and space, we collected small-volume seawater samples during the day and night over a 72 hour period from three locations that differed in spatial distance from 5 *Porites astreoides* coral colonies on a shallow reef in St. John, U.S. Virgin Islands: near-coral (sampled 5 cm horizontally from each colony), reef-depth (sampled 2 m above each colony) and surface seawater (sampled 1 m from the seawater surface). At all time points and locations, we quantified abundances of microbial cells, sequenced small subunit rRNA genes of bacterial and archaeal communities, and measured inorganic nutrient concentrations. *Prochlorococcus* and *Synechococcus* cells were consistently elevated at night compared to day and these abundances changed over time, corresponding with temperature, nitrite, and silicate concentrations. During the day, bacterial and archaeal alpha diversity was significantly higher in reef-depth and near-coral seawater compared to the surface seawater, signifying that the reef influences the diversity of the seawater microorganisms. At night, alpha diversity decreased across all samples, suggesting that photosynthesis may favor a more taxonomically diverse community. While *Prochlorococcus* exhibited consistent temporal rhythmicity, additional taxa were enriched in reef seawater at night compared to day or in reef-depth compared to surface seawater based on their normalized sequence counts. There were some significant differences in nutrient concentrations and cell abundances between reef-depth and near-coral seawater but no clear trends. This study demonstrates that temporal variation supersedes small-scale spatial variation in proximity to corals in reef seawater microbial communities. As coral reefs continue to change in benthic composition worldwide, monitoring microbial composition in response to temporal changes and environmental fluctuations will help discern normal variability from longer lasting changes attributed to anthropogenic stressors and global climate change.

## Introduction

Microorganisms play fundamental roles in coral reef ecosystem nutrient cycling [1, 2]. Microbial processes on coral reefs take place both in the reef benthos and within the water column. In the water column, picocyanobacteria and eukaryotic phytoplankton fix carbon into biomass through the process of photosynthesis. A significant fraction of this photosynthetically fixed carbon is released from cells through grazing, viral lysis, or exudation [reviewed by 3]. Heterotrophic bacteria in the water column respire organic matter released through all these processes and recycle inorganic nutrients back into the dissolved phase [4–6]. Within the reef benthos, symbiotic dinoflagellates residing within corals translocate photosynthate to the host and corals use this photosynthate for their own metabolisms, exuding dissolved and particulate organic matter (OM) into the water column [7–10]. Macroalgae and other benthic organisms are also sources of dissolved OM [4, 11]. Additionally, microorganisms within coral reef sediments mineralize carbon, fix nitrogen, and photosynthesize depending on their location in the sediment and the presence of oxygen [12, 13].

Most of our knowledge of reef seawater microbial community composition and function is obtained from opportunistic field sampling of reef seawater collected across reefs at a single point in time. This sampling approach has contributed knowledge about coral reef seawater microbial dynamics along various environmental and anthropogenic gradients [14–16], but does not allow for an understanding of resilience, resistance, and/or change in the same communities over time. Currently, coral reef ecosystems are experiencing dramatic shifts in reef composition [17], fish biomass [18] and nutrient availability [reviewed within 19] due to a variety of human-induced stressors including global climate change, pollution, coastal development, and overfishing. There is also evidence that a corresponding trophic shift is occurring within the microbial loop in coral reef ecosystems, favoring microbial communities that are dominated by more copiotrophic heterotrophic bacteria and potentially pathogenic taxa [20]. Due to the lack of microbial time-series studies and the observed trophic shifts in coral reef ecosystems, we have a limited understanding of baseline temporal variability of microbial community composition and function, further complicating our ability to discern consistent and recurring variability from ecosystem shifts over longer timescales.

On coral reefs, there are significant temporal changes due to the availability of light (influencing primary production), influences of tides, and diel vertical migration and grazing by zooplankton [21]. The availability of light is a major driver of net community metabolism on reefs, leading to primary production during the day and respiration at night [22, 23]. This metabolic switch also influences pH, oxygen concentrations, and aragonite saturation state within the seawater over a diel cycle [22–24]. Additionally, cyanobacterial mats on reefs release a significant amount of dissolved organic carbon into the water column at night [25]. Bearing in mind the diel fluctuations in light availability, net community metabolism, and grazing, as well as other processes on a reef, we would expect corresponding changes in microbial community dynamics across the reef.

Despite these diel fluctuations, there are only a handful of studies that have catalogued changes in microbial communities over the diel cycle. For example, the microbial community diversity in seawater sampled over a reef flat and adjacent to colonies of *Acropora formosa* changed between day and night [26]. In contrast, seawater microbial communities sampled adjacent to *Mussimillia braziliensis* showed no significant changes in composition or functional potential over a diurnal cycle spanning 48 hours [27]. It is surprising that neither of these studies reported changes in abundances of photosynthetic picocyanobacteria between day and night, considering the prevalence of these cells on some coral reefs [28] and their diel fluctuations in cell abundance in oligotrophic tropical ocean gyres [29]. A more recent study of seawater microbial communities sampled from forereefs in the Pacific Ocean detected synchronous changes in microbial community composition and function over one diel cycle [30]. They found consistent enrichment of specific taxa during both day and night and more genes indicative of diverse strategies for carbohydrate metabolism and general catabolism at night [30], demonstrating a shift in net metabolism of the collective microbial community over a diel cycle. That being said, no studies have tracked changes in reef seawater microbial community composition over a longer diel time-series, making it difficult to assess consistent diel and daily shifts in microbial biomass and community composition over time.

Reef seawater microbial communities can also vary in cell biomass, community composition, potential function, and growth dynamics based on water depth and proximity to reef organisms [31–33]. Seawater microbial communities located adjacent to corals are exposed to slightly different environmental and nutrient conditions as a result of exudation of organic matter and mucus from corals [4, 8], local changes in temperature, light availability [34], and water flow close to coral colonies [35]. These conditions may impact microbial community composition as well as potential microbial functions in the seawater surrounding corals at the scale of the momentum boundary layer [27, 32, 36], a layer of water surrounding the coral that is influenced by coral morphology and micro-currents caused by animal activity within the coral [35]. In fact, there is evidence that distinct microbial environments exist within 30 cm surrounding coral colonies in an environment called the coral ecosphere [37]. For example, coral ecosphere microbial communities were generally enriched with copiotrophic Gammaproteobacteria compared to microbial communities sampled from water >1 meter above the reef [37] and this finding corroborated earlier observations of copiotrophic enrichment in the seawater adjacent to corals [32]. Additionally, the Gammaproteobacteria *Endozoicomonas*, a ubiquitous coral tissue and mucus symbiont, was enriched within the ecosphere surrounding *P*. *astreoides*, indicating that the ecosphere environment may serve as a reservoir for coral symbionts and pathogens [37]. Furthermore, environmental conditions in the seawater surrounding corals also change at night due to decreased oxygen and pH in the diffusive boundary layer [38, 39], decreased exudation of dissolved organic matter (DOM) [40], and heterotrophic feeding by some coral species [reviewed within 41]. Diel changes in these conditions may lead to diel changes in composition that are unique to coral ecosphere microbial communities compared to communities sampled from seawater further away.

The present study was designed to compare diel, daily, and spatial variations in microbial cell abundances, inorganic macronutrient concentrations, and microbial community diversity and composition over the course of three days across three different environments including surface, reef-depth, and near-coral (5 cm away from individual coral colonies; coral ecosphere) seawater. We hypothesized that overall community composition would change temporally over diel and daily timescales. Additionally, we expected that coral ecosphere microbial communities would be enriched with Gammaproteobacteria compared to reef-depth and surface seawater communities and that these communities would shift over a diel cycle in relation to potential changes in environmental conditions close to the coral surface.

## Materials and methods

### Sample collection

**Fig 1:**
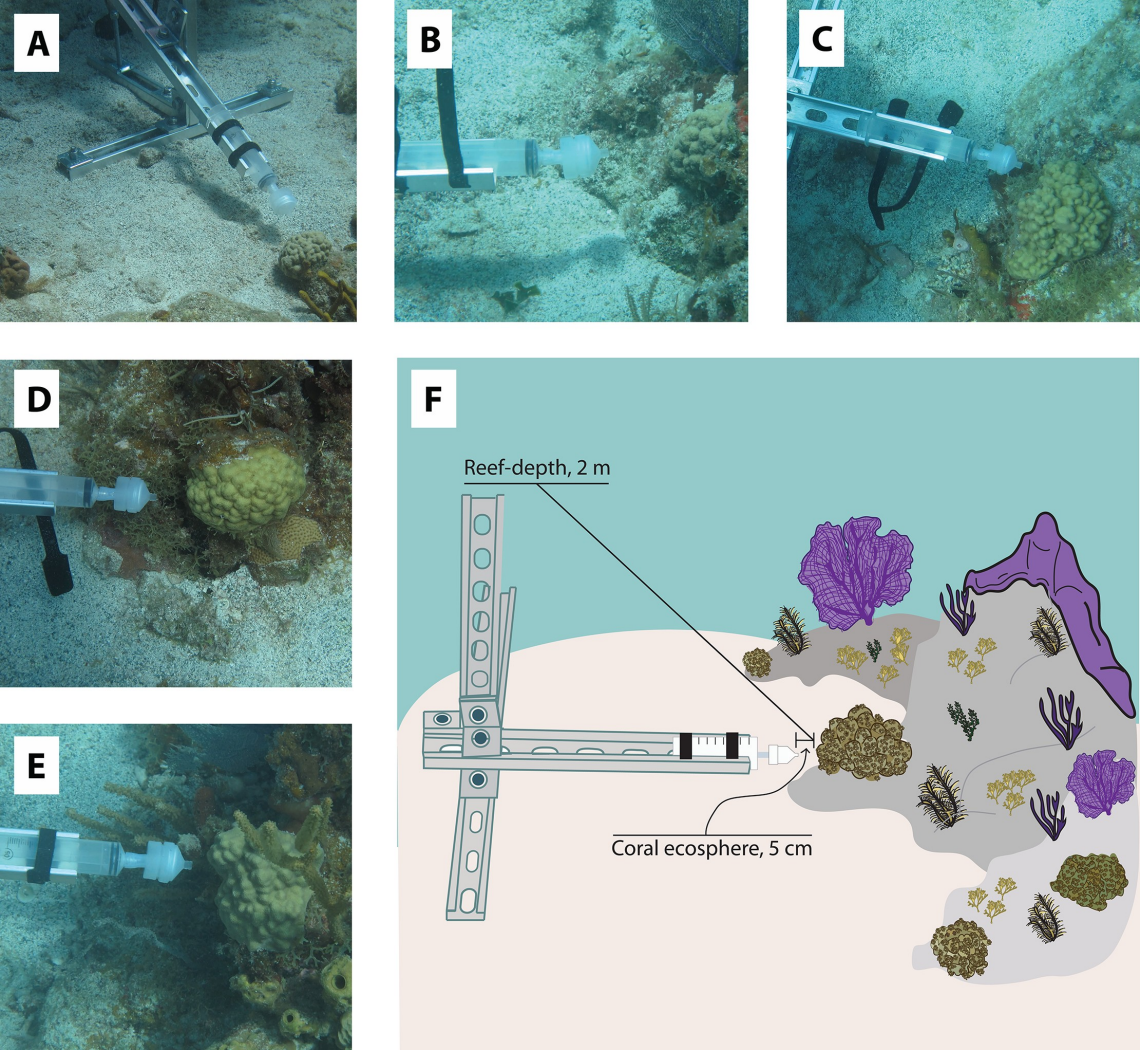
Photographs of the selected *P*. *astreoides* coral colonies located adjacent to deployed coral ecosphere sampling devices (A-E) and a sampling diagram detailing seawater sampling locations from coral ecosphere and reef-depths (F). Diagram is not drawn to scale.

This study was conducted under the United States National Park Services permit: VIIS-2017-SCI-1109. Five *Porites astreoides* colonies and one sand patch were selected and marked with flagging tape by divers on Ram Head reef (18°18’07.3” N, 64°42’14.5” W; 8 m depth in sand) in St. John, U. S. Virgin Islands. Colonies of various sizes (3–16 inches in diameter) from a range of heights above the sea floor (1–27 cm) were selected and these colonies were labeled A through E (Fig 1). Additionally, colonies were evenly distributed across the reef in order to minimize location effects (range of 3.6 to 14 meters between each colony). All colonies were located directly next to sand patches based on colony size constraints and the space needed for deployment of the custom made Coral Ecosphere Sampling Devices (CESD) (Fig 1). Six CESD made out of aluminum strut material were deployed adjacent to each sampling location with sand screws. The last CESD was placed in a wide sand patch with no corals or benthic organisms located in its vicinity and this sampling location was used as a ‘no-coral’ control. Divers positioned the CESD so that a 60 ml syringe with an attached filter holder could be placed 5 cm away from the middle of the colony (Fig 1). Light and temperature loggers (8K HOBO/PAR loggers; Onset, Wareham, MA) were zip-tied to the end of each CESD and programmed to collect temperature and relative light intensity measurements every 5 minutes over the course of the three day study.

An hour after CESD deployment, scuba divers collected the first set of samples (Day 1, 3:00 pm). Filter holders were pre-loaded with 0.22 μm pore size Supor^**®**^ filters (Pall Corporation, Ann Arbor, MI, USA) and were contained within sterile Whirl-pack^**®**^ bags prior to sampling. Divers also descended with acid-washed polyethylene nutrient bottles (30 ml volume) to collect seawater samples for unfiltered inorganic nutrient analysis and flow cytometry. Samples were also collected for analysis of total organic carbon, but are not included in this study because they became contaminated during sample storage. At depth, seawater samples (60 ml) collected for amplicon-based microbial community analyses were taken at 2 different stationary locations relative to the CESD device (with the exception of collections completed at the sand-patch location). Reef-depth samples were collected first at the top of the CESD (2 m from the colony) in order to minimize stirring close to the coral ecosphere sampling area (Fig 1). To collect the sample, a diver attached a piece of acid-cleaned Masterflex silicone tubing to connect the end of the filter holder to the mouth of the syringe and then used reverse filtration to pull seawater through the filter. The filter-holder was then placed in an individual Whirl-pack^**®**^ bag and sealed. After collection of microbial biomass with the syringe, a nutrient sample was collected. After collection of the reef-depth sample, a diver attached the filter holder to the syringe, slowly descended closer to the coral colony, but behind the CESD to maintain sufficient distance from the sampling area, and then placed the syringe into the syringe holder located on the horizontal arm of the CESD. As before, the diver first collected the coral ecosphere sample (5 cm from the colony) onto the filter followed by a nutrient sample in the same location (Fig 1). Replicate samples collected for microbial community analyses were collected from both seawater environments surrounding each colony on the first dive, but not were not collected on the following dives due to time constraints. Surface seawater samples (< 1 m) were collected using 60 mL syringes at each time point from the dive boat.

This sampling scheme was repeated at approximately 3 am and 3 pm for the next three days, totaling up to 6 sampling time points. Divers sampled each colony and collected samples in the same order (reef-depth followed by coral ecosphere) during all time points. After collection, samples were placed in a cooler equipped with blue-ice packs for the transit from the reef to the lab and then samples were processed immediately. Over the course of sampling, 85 seawater samples for microbial community analyses were collected.

After the last time point, coral tissue was collected from each colony (close to the area where the coral ecosphere seawater was sampled) using a hammer and chisel and the CESD were removed. Sand was also collected in the location where the sand control CESD device was deployed.

### Sample processing

In the laboratory, sterile syringes were used to remove residual seawater trapped within filter holders and then filters were placed into cryovials, flash-frozen in a dry shipper charged with liquid nitrogen, and then transferred into a -20° C freezer. Seawater collected for flow cytometric analysis was subsampled from unfiltered nutrient samples and preserved with paraformaldehyde (Electron Microscopy Sciences, Allentown, PA) to a final concentration of 1% (by volume). Nutrient, DNA, and flow cytometry samples were shipped frozen back to Woods Hole Oceanographic Institution and ultimately stored at -80°C prior to analysis. The coral tissue and sand samples were stored in a second dry shipper and ultimately at -80°C until they were processed.

### Macronutrient analysis and flow cytometry

Frozen and unfiltered nutrient samples were analyzed with a continuous segmented flow-system using previously described methods [as in 42]. The concentrations of nitrite + nitrate, nitrite, phosphate, ammonium, and silicate were measured in all of the samples. Nitrate concentrations were obtained by subtracting the nitrite concentration from the nitrite + nitrate measurements for each sample.

Samples collected for flow cytometry were analyzed using colinear analysis (laser excitation wavelength of 488 nm, UV) on an Altra flow cytometer (Beckman Coulter, Pasadena, CA). Unstained subsamples were used to enumerate the abundances of picocyanobacteria (*Prochlorococcus*, *Synechococcus*) and picoeukaryotes. Subsamples were also stained with Hoechst’s stain (1 μg ml^-1^ final concentration) in order to estimate the abundance of unpigmented cells (an estimate of heterotrophic bacterial abundance) in the samples [43]. To determine the abundance of unpigmented cells, picocyanobacterial and picoeukaryotic cells were subtracted from stained cell abundances (representing all cells containing DNA) per each sample. FlowJo (v. 6.4.7) software was used to estimate the abundance of each cell type. The abundance of total cells was calculated by adding the cell counts obtained for each of the respective picoplankton classes together for each sample.

### DNA extraction, amplification, pooling, and sequencing

DNA was extracted from filters using a sucrose-lysis extraction method and Qiagen spin-columns [44]. Control extractions were also completed with blank filters (filters without biomass) in order to account for contamination from the filters or extraction reagents. Lastly, diluted DNA from a synthetic staggered mock community (BEI Resources, NIAID, NIH, Manassas, VA, USA, as part of the Human Microbiome Project: Genomic DNA from Microbial Mock Community B (staggered, low concentration), v5.2 L, for 16S rRNA Gene Sequencing, HM-783D) was used to account for amplification and sequencing errors in downstream microbial community analyses. Coral tissue was removed from the skeleton using air-brushing with autoclaved 1% phosphate-buffered-saline solution [45, 46]. The coral tissue slurry was pelleted using a centrifuge and the phosphate-buffered-saline supernatant was discarded. DNA was extracted from each pellet (300 mg of tissue) using a modified version of the DNeasy DNA extraction kit protocol (Qiagen, Germantown, MD). The lysis buffer in the kit was added to each tube followed by approximately 300 mg of garnet beads and 300 mg of Lysing B matrix beads (MP Biomedicals, Solon, OH). The tubes were subjected to a bead-beating step for 15 minutes so that the beads could break up the coral tissue [46]. After bead-beating, 20 μl of proteinase-k was added to each tube and the samples were incubated with gentle agitation for 10 minutes at 56°C. After these modifications, the DNeasy protocol (Qiagen) was followed to complete extractions.

Extracts were amplified with barcoded primers 515FY and 806RB targeting the V4 hypervariable region of the bacterial and archaeal small subunit (SSU) ribosomal RNA gene [47, 48]. The forward primer: 5’ TATGGTAATTGTGTGYCAGCMGCCGCGGTAA 3’ [47] and reverse primer: 3’ AGTCAGTCAGCCGGACTACNVGGGTWTCTAAT 5’ [48] were used, along with the barcodes, to amplify and tag each sample prior to pooling. We used forward and reverse primers with degeneracies in order to eliminate amplification biases against Crenarchaeota/ Thaumarchaeota [47] and SAR 11 [48]. Triplicate polymerase chain reactions (25 μl volume) were run with 2 μl of DNA template from each sample using the same barcodes in order to minimize the formation of chimeras during amplification. The reaction conditions included: a 2 minute hot start at 95°C followed by 36 cycles of 95°C for 20 seconds, 55°C for 15 seconds, and 72°C for 5 minutes. The final extension step was 72°C for 10 minutes. Triplicate barcoded amplicons were pooled and screened using gel electrophoresis to assess quality and amplicon size. Amplicons were purified using the MinElute Gel Extraction Kit (Qiagen) and pooled to form the sequencing library. The library was sequenced (paired-end 2x250 bp) at the Georgia Genomics and Bioinformatics Core with a Miseq (Illumina, San Diego, CA) sequencer and raw sequence reads are available at the NCBI Sequence Read Archive under BioProject # PRJNA550343.

### Microbial community analyses

Raw sequences were quality-filtered and grouped into amplicon sequence variants (ASVs) using DADA2 [49]. Reads were filtered by removing sequences with any Ns, sequences with quality scores less than 2, residual phiX sequences, and reads with expected errors higher than 2 (maxN = 0, truncQ = 2, rm.phix = TRUE, and maxEE = 2). Reads were then trimmed and dereplicated. The DADA2 algorithm was used to infer the number of different ASVs (8,357 distinct ASVs), paired reads were merged, an ASV table was constructed, and chimeras were removed (1% of all ASVs). Taxonomy was assigned to each ASV using the Silva v.132 reference database with the Ribosomal Database Project naïve bayesian classifier [50] and exact matching between ASVs and sequenced strains [51]. The mock communities were used to assess the performance of the program as well as sequencing error rates. DADA2 inferred 15, 17, and 17 strains within the mock community (compared to the 20 expected stains present at different concentrations within the staggered community) and 13, 14, and 14 of the strains were exact matches to the expected sequences from the mock community reference file. Sequence recovery was slightly lower than expected, but is comparable to normal performance of DADA2 on this staggered mock community [49].

The R packages Phyloseq [52], Vegan [53], DESeq2 [54], and ggplot2 [55] were used for downstream analyses of the microbial community. Samples with less than 1000 reads (2 samples) were removed. In addition, ASVs identifying as chloroplasts were removed. Sequences representing ASVs that identified as “NA” at the phylum level were checked using the SINA aligner and classifier (v.1.2.11) [56] and then removed if not identified as bacteria or archaea at 70% similarity. The average number of reads across all seawater samples used in microbial community analyses was 58,398 (± 32,184 standard deviation) with a range of 11,502–206,689 reads. The average number of reads in coral tissue samples was 38,096 (± 23,854) with a range of 11,538–59,437 reads. DNA extraction control communities were initially inspected and then removed because they fell out as outliers compared to the highly similar seawater microbial communities.

Sequences were subsampled to 11,502 sequences per sample with replacement prior to alpha diversity analyses to minimize the influence of differential sequence depth. For the remaining analyses, relative abundances of non-subsampled data were used except for DESeq2 (see below). Taxonomic bar plots and metrics of alpha diversity (observed richness of ASVs and Shannon’s diversity index) were made and calculated using Phyloseq. Constrained analysis of principal coordinates (CAP) based on Bray–Curtis dissimilarity was completed (using ‘capscale’ in Vegan) and variance partitioning was used to identify which of the measured environmental parameters significantly (p < 0.01) contributed to shifts in the ecosphere and reef-depth seawater microbial community composition over time. To complete variance partitioning, the function ‘ordistep’ (in Vegan) was used to select a subset of potentially significant variables and the function ‘varpart’ was then used to complete variance partitioning via redundancy analysis ordination. Finally, the significance of each individual variable was tested by passing the ‘rda’ function to individual ANOVA tests (permutations = 999). Surface seawater samples were omitted from the CAP analysis because corresponding nutrient, physicochemical, and cell abundance data was not collected from surface seawater. Permutational multivariate analysis of variance using distance matrices (PERMANOVA/Adonis) tests identified categorical factors that significantly (p<0.05) contributed to the similarity between the microbial communities. Surface seawater samples were also excluded from this analysis for the same reasons stated above.

DESeq2 was used to identify differentially abundant ASVs between day and night as well as reef-associated (reef-depth and coral ecosphere) compared to surface microbial communities (using the “local” fitType parameter to estimate gene dispersion). Prior to testing differential enrichment, ASV counts were normalized using mean ratios based on a negative binomial distribution [54, 57]. Lastly, the Rhythmicity Analysis Incorporating Non-parametric methods (RAIN) R package was used to identify ASVs that experienced rhythmic change in relative abundance over a period of 24 hours [58]. This approach implements a nonparametric method to detect symmetric and nonsymmetric rhythms in the data. Briefly, the relative abundances of each ASV are separated by time-point and location within the oscillation period and are compared separately using k-sample rank tests for umbrella alternatives [58, 59]. RAIN analysis was completed separately for reef-depth and coral ecosphere seawater and the input ASV matrix for the RAIN analysis was center log-ratio transformed and detrended following previous methods [60]. Only ASVs with significant p-values (p < 0.05) after adaptive Benjamini-Hochberg correction were reported to control for false recovery rates [61]. Sequence counts were converted into relative abundances for all microbial community analyses, except for calculating metrics of alpha diversity and conducting the DESeq2 procedure.

### Additional statistical analyses

A principal components analysis (PCA) was completed on the original data in order to summarize changes in picoplankton abundances, inorganic nutrient concentrations, and relative light and temperature information collected from the HOBO loggers and reduce the dimensionality of this data. Separate PCAs were also generated using samples collected during either day or night to observe trends specific to these times. PERMANOVA tests were conducted on the original picoplankton cell abundances and macronutrient concentrations converted into Bray-Curtis dissimilarity indices in order to investigate broader changes in these parameters across the factors of interest (diel, day, colony, and distance) (S1 and S2 Tables). Macronutrient concentrations, cell abundances, and metrics of bacterial and archaeal alpha diversity were inspected for normality using Shapiro-Wilks tests. Log_10_ transformations and/or removal of extreme outliers were used to normalize a majority of the data that was not already normally distributed. Outliers were identified using boxplots and removed if they were more than 1.5 times the interquartile range above the third or below the first quartile for each diel and day grouping. Normally distributed data was then subjected to 4-way ANOVA tests followed by post-hoc Tukey’s honest significant difference (HSD) multiple comparison tests (95% family-wise confidence level) to test for significant differences between factors. Using this test, pairwise comparisons are conducted and p-values are corrected for multiple comparisons to avoid making Type I errors. Residuals of the ANOVA tests were also inspected for normality using quantile-quantile plots. Project data can be accessed using the BCO-DMO repository (doi:10.1575/1912/bco-dmo.775229.1).

## Results

### Picoplankton abundances and inorganic macronutrient concentrations

**Fig 2:**
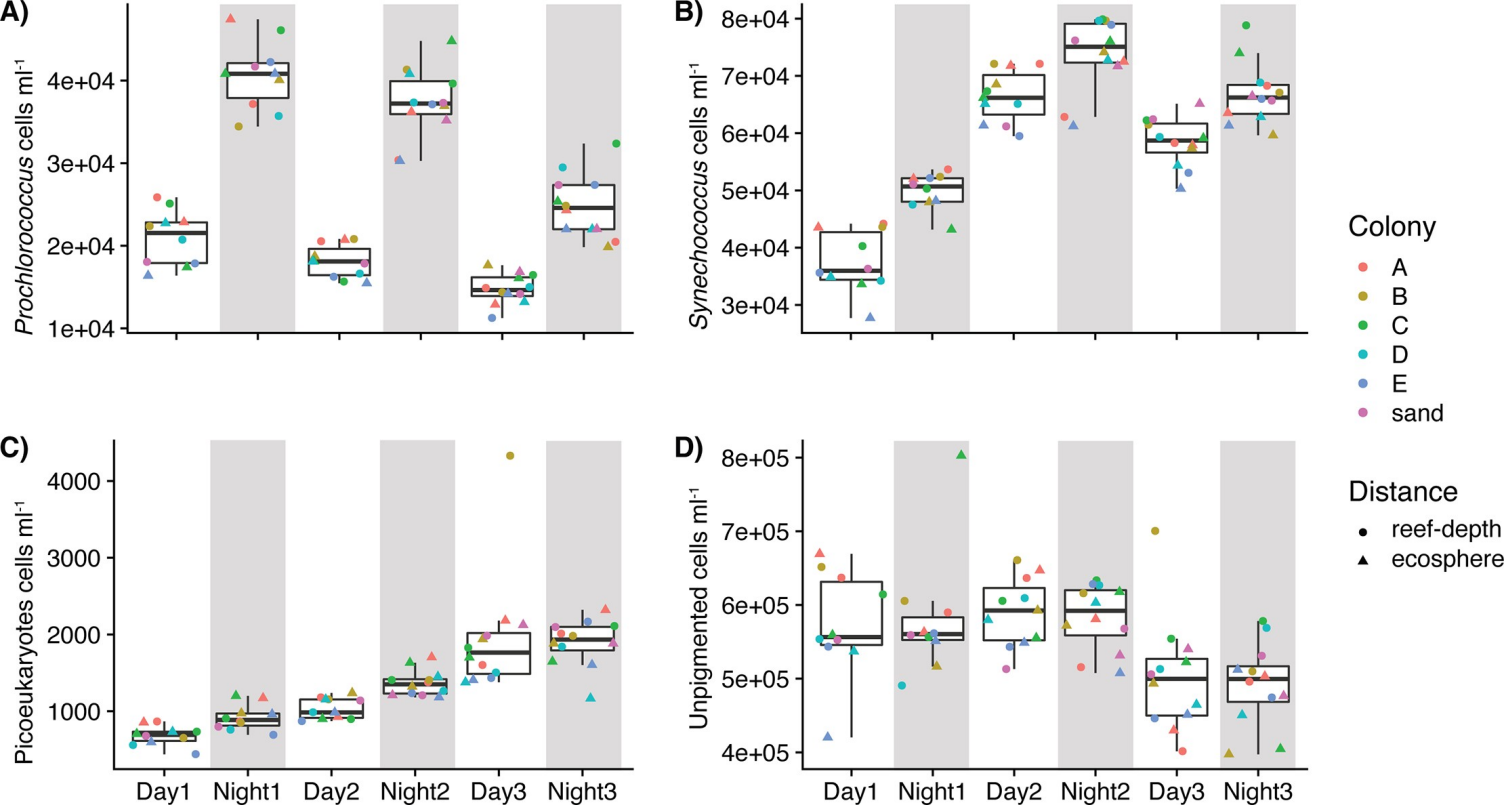
Comparison of picoplankton cell abundances over three consecutive days for A) *Prochlorococcus*, B) *Synechococcus*, C) picoeukaryotes, and D) unpigmented cells (generally heterotrophic bacteria and archaea). Each point represents a sample. Point shape corresponds to sampling distance from the coral and point color reflects the colony adjacent to where sampling was conducted. Gray shading indicates samples collected at night. Lower and upper edges of the boxplot correspond to the first and third quartiles, the whiskers extend to the largest or smallest value at 1.5 times the interquartile, and the black bar across the box represents the median. Original, untransformed cell counts are presented.

Overall, picoplankton abundances did not significantly differ between coral ecosphere and reef-depth seawater (PERMANOVA, p = 0.171, S1 Table), but changed between day and night, colony, and/or over the course of the three-day study depending on the specific group (Fig 2, Table 1 and S1 Table). *Prochlorococcus* and *Synechoccocus* populations showed the strongest diel signal with abundances increasing significantly at night, compared to abundances measured the previous day (Fig 2A and 2B, Table 1). *Prochlorococcus* consistently doubled at night relative to day, but decreased throughout the study (Fig 2A). Over the course of the study, day and night abundances of *Synechoccocus* and picoeukaryotes increased significantly compared to their initial abundances (Fig 2B and 2C, Table 1, Tukey’s test, adjusted p-value < 0.05). The abundances of unpigmented cells were generally similar between day and night, but experienced significant changes by day (Fig 2D, Table 1). The abundance of unpigmented cells was significantly lower on day 3 compared to days 1 and 2 (Tukey’s test, adjusted p-value < 0.05). Additionally, there was more spatial variability in the abundances of *Prochlorococcus*, *Synechoccocus*, and unpigmented cells compared to picoeukaryotes across the reef at each time point. For *Prochlorococcus* and unpigmented cells, there were no significant trends in abundance by colony/sand (A-E, F) (Table 1). However, picoeukaryotic cell abundances were significantly different (Table 1) between coral colonies D and A (Tukey’s test, adjusted p-value = 0.03), E and A (Tukey’s test, adjusted p-value = 0.007), D and B (Tukey’s test, adjusted p-value = 0.02), and E and B (Tukey’s test, adjusted p-value = 0.005). Additionally, *Synechococcus* abundance was significantly lower (Table 1) in reef-depth and ecosphere seawater surrounding colony E compared to colonies A (Tukey’s test, adjusted p-value = 0.01), B (Tukey’s test, adjusted p-value = 0.02), and C (Tukey’s test, adjusted p-value = 0.02).

**Table 1:** Summary of 4-way ANOVA statistical tests presenting results for the four main factors and any significant interactions between factors.

Parameter	Factor	Df	Sum sq	Mean Sq	F-value	p-value
*Prochlorococcus* cell abundance	Diel*	1	1.28	1.28	457.26	< 0.05
	Day*	2	0.41	0.20	73.22	< 0.05
	Distance	1	0.0012	0.0012	0.44	0.51
	Colony	5	0.029	0.0059	2.098	0.081
	Diel: day*	2	0.026	0.013	4.63	< 0.05
	Diel: day: distance*	2	0.018	0.0092	3.28	< 0.05
*Synechococcus* cell abundance	Diel*	1	0.0100	0.0100	86.15	< 0.05
	Day*	2	0.51	0.26	220.78	< 0.05
	Distance*	1	0.011	0.010	9.028	< 0.05
	Colony*	5	0.022	0.0045	3.87	< 0.05
	Diel: day*	2	0.019	0.0096	8.26	< 0.05
Picoeukaryotic cell abundance	Diel*	1	0.11	0.11	20.74	< 0.05
	Day*	2	1.56	0.78	148.43	< 0.05
	Distance	1	0.0014	0.0014	0.26	0.61
	Colony*	5	0.13	0.026	4.984	< 0.05
	Diel: day*	2	0.051	0.026	4.886	< 0.05
	Day: distance*	2	0.058	0.029	5.468	< 0.05
Unpigmented cell abundance	Diel	1	1.64e+08	1.64e+08	0.043	0.084
	Day*	2	1.13e+11	5.64e+10	14.68	< 0.05
	Distance	1	1.10e+10	1.10e+10	2.87	0.096
	Colony	5	3.69e+10	7.38e+09	1.92	0.11
Phosphate concentration	Diel*	1	0.079	0.079	43.45	< 0.05
	Day*	2	0.043	0.022	11.92	< 0.05
	Distance*	1	0.010	0.010	5.59	< 0.05
	Colony	5	0.0076	0.0015	0.84	0.53
	Diel: day*	2	0.021	0.010	5.75	< 0.05
	Diel: distance*	1	0.019	0.019	10.22	< 0.05
Silicate concentration	Diel	1	0.00003	0.00003	0.045	0.83
	Day*	2	0.069	0.035	53.52	< 0.05
	Distance	1	0.00037	0.00037	0.58	0.45
	Colony	5	0.0040	0.00080	1.23	0.31
	Diel: day*	2	0.019	0.0093	14.37	< 0.05
Ammonium concentration	Diel*	1	0.18	0.18	21.67	< 0.05
	Day*	2	0.26	0.13	15.14	< 0.05
	Distance*	1	0.068	0.068	7.98	< 0.05
	Colony	5	0.090	0.018	2.10	0.083
	Diel: day*	2	0.089	0.045	5.24	< 0.05
Nitrate concentration	Diel*	1	0.028	0.028	6.073	< 0.05
	Day*	2	0.094	0.047	10.25	< 0.05
	Distance	1	0.0028	0.0028	0.62	0.44
	Colony	5	0.032	0.0064	1.39	0.24
	Diel: day*	2	0.054	0.027	5.84	< 0.05
Nitrite concentration	Diel	1	0.00013	0.00013	0.33	0.568
	Day	2	0.0018	0.00091	2.38	0.102
	Distance	1	0.000001	0.0000007	0.002	0.966
	Colony	5	0.0025	0.00050	1.32	0.272
Bacterial and Archaeal Observed Richness	Diel*	1	0.45	0.45	34.56	< 0.05
	Day*	2	0.29	0.14	11.15	< 0.05
	Sample type*	2	0.72	0.36	27.93	< 0.05
	Colony	5	0.14	0.028	2.14	0.074
Bacterial and Archaeal Shannon’s Diversity	Diel*	1	1.38	1.38	11.58	< 0.05
	Day*	2	4.70	2.35	19.73	< 0.05
	Sample type*	2	2.41	1.21	10.14	< 0.05
	Colony*	5	2.061	0.41	3.46	< 0.05

*indicates variable is significantly different by that factor

**Fig 3:**
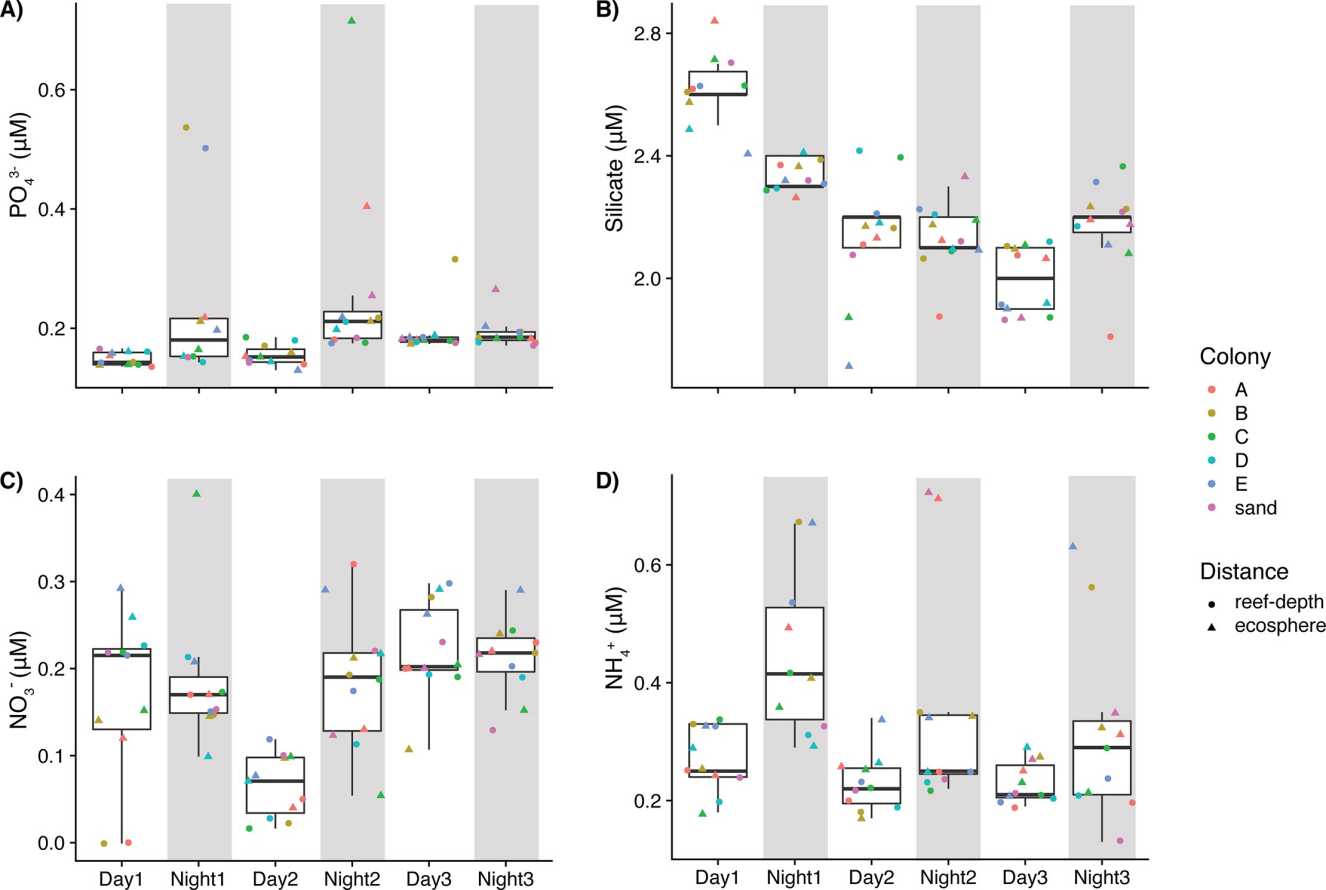
Comparison of inorganic nutrient concentrations over three days for A) phosphate (PO_4_^3-^), B) silicate, C) nitrate (NO_3_^-^), and D) ammonium (NH_4_^+^). Each point represents a sample. Point shape corresponds to sampling distance from the coral and point color reflects the colony adjacent to where sampling was conducted. Gray shading indicates samples collected at night. Lower and upper edges of the boxplot correspond to the first and third quartiles, the whiskers extend to the largest or smallest value at 1.5 times the interquartile, and the black bar across the box represents the median. Untransformed concentrations and their outliers are presented.

The concentrations of inorganic macronutrients were low and generally exhibited fluctuations between day and night as well as over time (Fig 3, Table 1 and S2 Table). Phosphate, ammonium, and nitrate all increased significantly at night compared to day and silicate concentrations followed the opposite trend (Table 1). All nutrient concentrations with the exception of nitrite underwent significant daily changes: phosphate was significantly lower on day 1 compared to days 2 and 3 (Tukey’s test, adjusted p-value < 0.05), nitrate was significantly higher on days 1 and 3 compared to day 2 (Tukey’s test, adjusted p-value < 0.05), silicate was significantly lower on days 2 and 3 compared to day 1 (Tukey’s test, adjusted p-value < 0.05), and ammonium was significantly higher on day 1 compared to days 2 and 3 (Tukey’s test, adjusted p-value < 0.05) (Fig 3, Table 1). Ammonium and phosphate concentrations were higher in ecosphere seawater compared to reef-depth seawater, but concentrations of the other macronutrients did not vary with spatial distance from the coral colonies (S1 Fig, Table 1).

**Fig 4:**
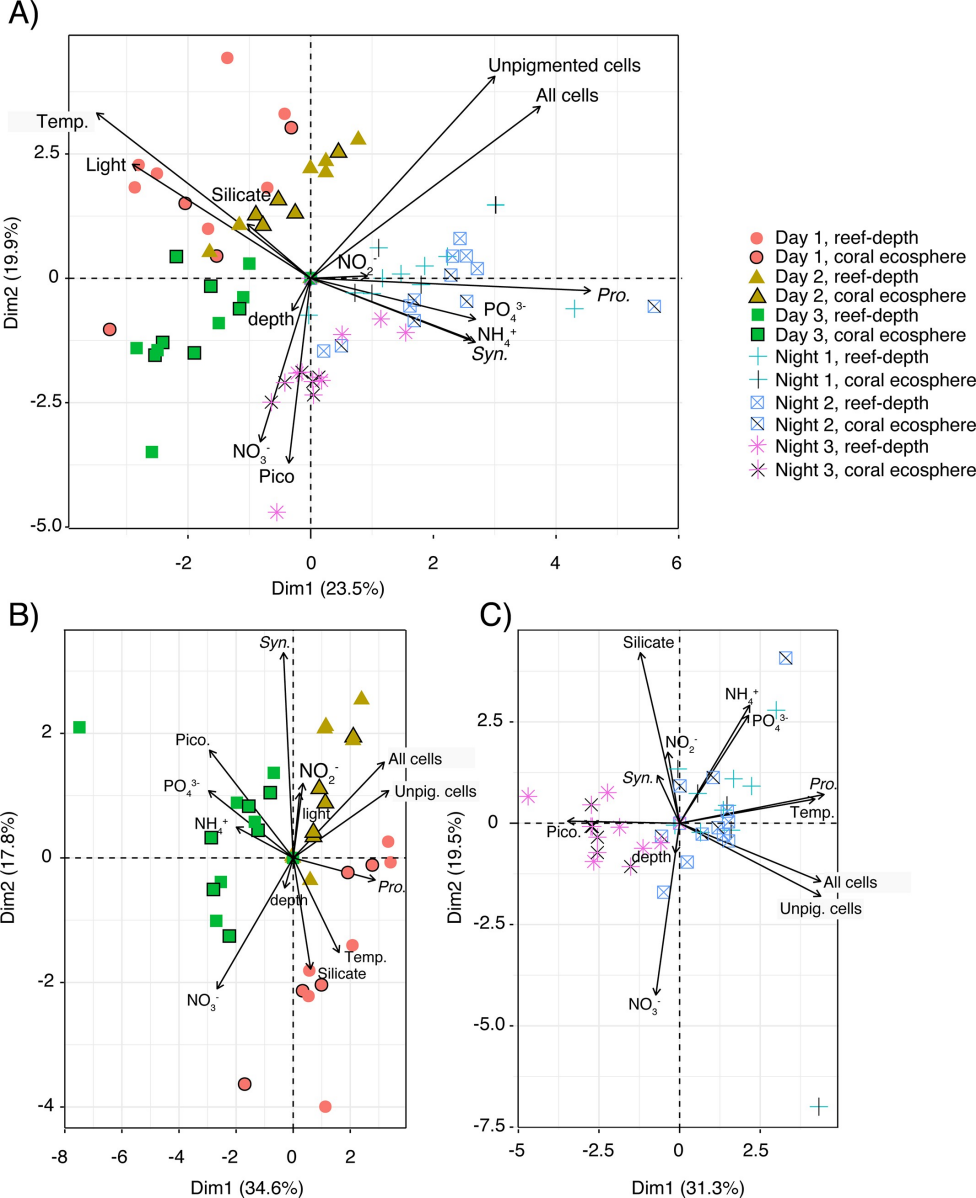
PCA biplots of picoplankton abundances, relative light, temperature, sampling depth, and inorganic macronutrient concentrations across A) all time-points, B) samples collected during the day, and C) samples collected during the night. Point color and shape reflect the day and time of sampling. Symbols outlined in black or with 1–2 black lines indicate ecosphere samples. Abbreviations are as follows: *Syn*. = *Synechococcus* cell abundance, *Pro*. = *Prochlorococcus* cell abundance, Pico. = picoeukaryote, Unpig. cells = unpigmented cells, and temp. = temperature.

PCA illustrated the diel signal of *Prochlorococcus* and *Synechoccocus* abundances, and increased temperature and relative light during the day (Fig 4). In addition, there was a correlation between nitrate concentrations and picoeukaryotic cells (Fig 4A). The PCA completed on samples collected during the night revealed correlations between *Prochlorococcus* abundances with temperature and *Synechococcus* abundances with nitrite and silicate concentrations (Fig 4C). Day and night samples from day 3 were each correlated with picoeukaryotes (Fig 4A and 4B).

### Microbial community alpha diversity

**Fig 5:**
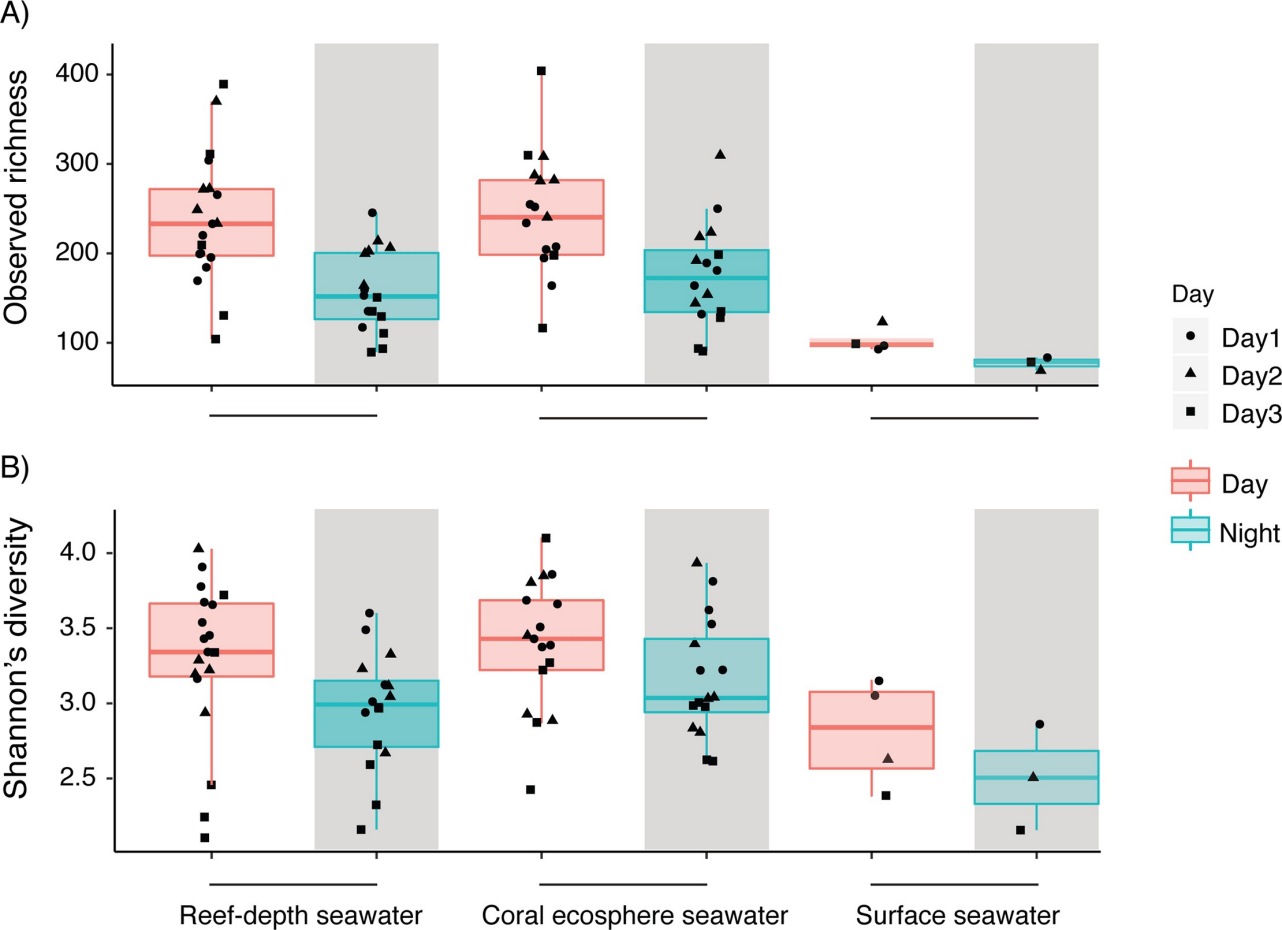
Alpha diversity metrics of the bacterial and archaeal community by sample type and time, based on SSU rRNA gene sequences grouped into ASVs and subsampled with replacement to 11,500 sequences per sample. Observed richness is indicated in A) and Shannon’s diversity is presented in B). Each point represents a sample. The date of sampling is denoted by the shape and the point color reflects day or night. Gray shading indicates samples collected at night. Lower and upper edges of the boxplot correspond to the first and third quartiles, the whiskers extend to the largest or smallest value at 1.5 times the interquartile, and the black bar across the box represents the median.

ANOVA testing revealed that diel changes, daily changes, and sample type significantly influenced observed bacterial and archaeal richness (Fig 5A, Table 1). Richness was higher during the day compared to night (Tukey’s test, adjusted p-value < 0.05) and richness was significantly higher on day 2 (Tukey’s test, adjusted p-value < 0.05). Richness was similar between reef-depth and coral ecosphere microbial communities (Tukey’s test, adjusted p-value = 0.67), although there was more variability across samples collected on Day 3 (Fig 5). The largest differences in observed richness occurred between the different sample types of reef-depth/ecosphere seawater and surface seawater (Tukey’s test, adjusted p-value < 0.05). Reef-depth seawater had significantly higher richness during the day compared to night (Fig 5A). Additionally, richness during the day in reef-depth and coral ecosphere samples was more variable compared to communities surveyed at night (Fig 5). Across all samples, daily observed richness was significantly different between days 1 and 2 (Tukey’s test, adjusted p-value < 0.05) and days 2 and 3 (Tukey’s test, adjusted p-value < 0.05). Overall, there was more variable richness in reef-depth and coral ecosphere seawater compared to surface seawater microbial communities (Fig 5). There were no differences in bacterial and archaeal richness sampled surrounding the different coral colonies or between the coral seawater and the sand control seawater (Table 1).

To account for both bacterial and archaeal community richness and evenness, the Shannon’s diversity index was computed. Shannon’s diversity followed the same general trends that were observed for richness, with diel changes, daily changes, sample type, and colony significantly influencing differences (Fig 5B, Table 1). Additionally, Shannon’s index was significantly different between coral colony A and colonies C (adjusted p-value = 0.017) and D (adjusted p-value = 0.010) in pair-wise coral colony contrasts using post-hoc Tukey’s tests.

### Microbial community composition

**Fig 6:**
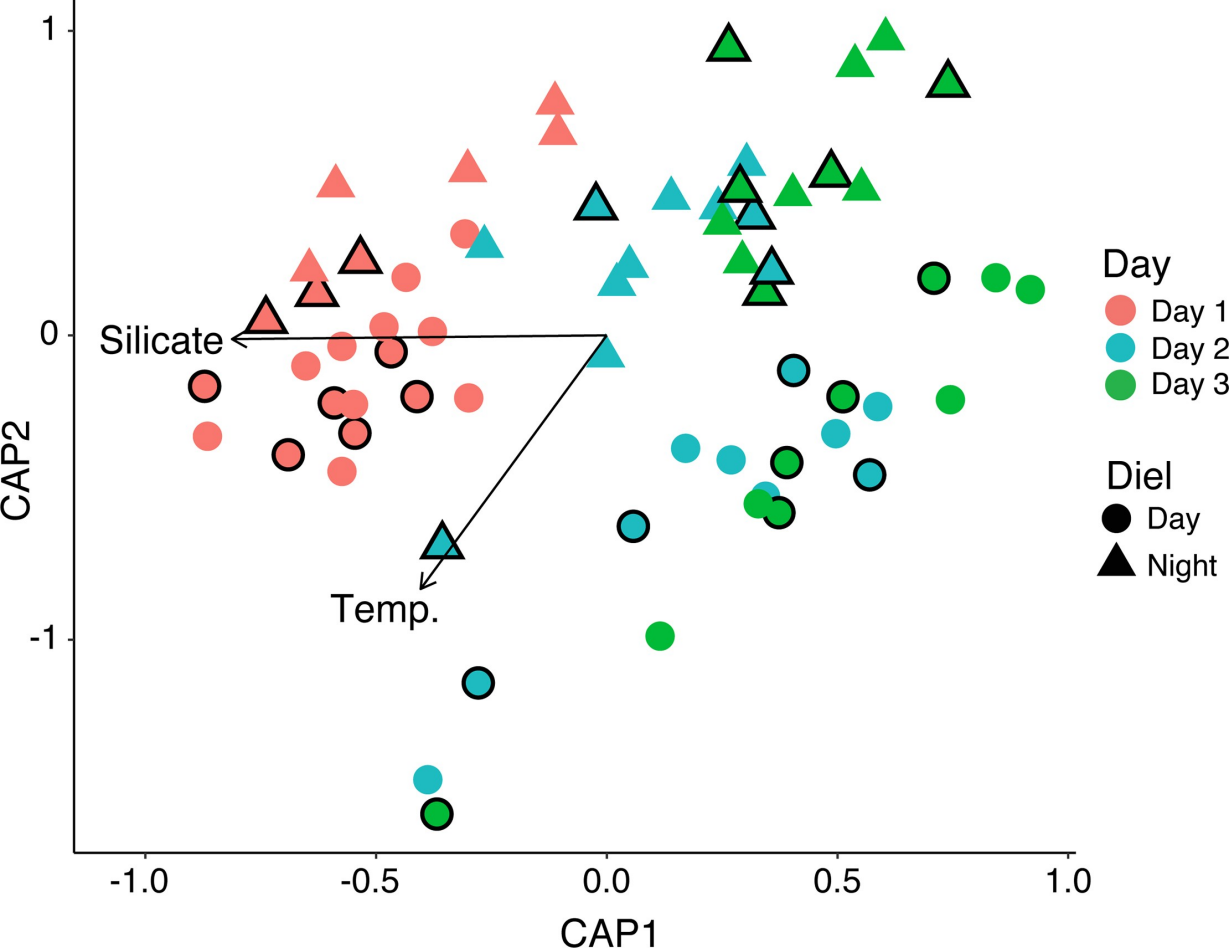
CAP of bacterial and archaeal SSU rRNA genes sequenced from reef-depth and coral ecosphere samples compared using the Bray–Curtis index. Significant environmental variables (ANOVA, p < 0.05) are overlaid on the plot as vectors and labeled. Symbols outlined in black indicate coral ecosphere samples.

Regardless of proximity to corals, reef seawater bacterial and archaeal compositions, assessed using SSU rRNA gene sequencing, were highly similar, but consistently shifted between day and night as well as over time (Fig 6). CAP of the Bray–Curtis dissimilarity matrix and variance partitioning of quantitative environmental variables revealed that silicate concentrations (ANOVA test F(1,61) = 7.59, p = 0.003) and temperature (ANOVA test, F(1,61) = 7.034, p = 0.019) significantly explained shifts in microbial community composition over time (Fig 6). The percentage of variation explained by silicate was greater (adjusted R^2^ = 0.30) compared to temperature (adjusted R^2^ = 0.087). In addition, communities sampled at the same time were most similar to each other, but also ordinated more broadly by day or night (Fig 6). A PERMANOVA (Adonis) test conducted on the Bray–Curtis dissimilarity matrix supported this observation by indicating that the categorical factors of day (day 1, 2, 3), and diel (day vs. night) significantly explained microbial community dissimilarity (Table 2). That being said, the PERMANOVA test also revealed that distance (5 cm vs. 2 m), colony (A-E and sand), and the interactions between a majority of the factors significantly explained microbial community dissimilarity (Table 2).

**Table 2:** Results of PERMANOVA (ADONIS) test examining factors influencing amplicon-based reef-depth and coral ecosphere seawater microbial community dissimilarity, using 999 permutations.

Factor	DF^$^	Sums of Squares	MeanSqs^§^	F model	R2	Pr(>F)^¶^
Diel*	1	0.12	0.12	9.10	0.070	0.001
Day^&^	2	0.58	0.29	41.69	0.33	0.001
Distance^	1	0.047	0.047	6.78	0.02	0.001
Colony^‡^	5	0.18	0.035	5.046	0.10	0.001
Diel: day	2	0.063	0.031	4.52	0.036	0.003
Diel: distance	1	0.012	0.012	1.79	0.0071	0.156
Day: distance	2	0.020	0.0098	1.40	0.011	0.239
Diel: colony	5	0.089	0.018	2.56	0.051	0.021
Day: colony	10	0.16	0.16	2.36	0.093	0.017
Distance: colony	5	0.058	0.012	1.68	0.033	0.115
Diel: day: distance	2	0.025	0.013	1.83	0.014	0.119
Diel: day: colony	10	0.19	0.019	2.74	0.11	0.005
Diel: distance: colony	4	0.046	0.012	1.67	0.026	0.119
Day: distance: colony	8	0.098	0.012	1.75	0.056	0.091
Diel: day: distance: colony	1	0.022	0.022	3.12	0.012	0.041
Residuals	6	0.042	0.0070		0.024	
Total	65	1.76			1.00	

* Day vs. night

^&^ Day 1, 2, or 3

^Reef-depth (2 m) or coral ecosphere (5 cm) sampling distances from the coral colony or sand

^‡^Coral colonies A-E or sand

^$^DF = degrees of freedom

^§^MeanSqs = mean squares

^¶^Pr(>F) = permutational p-values using pseudo-F ratios. Exact p-values are shown

**Fig 7:**
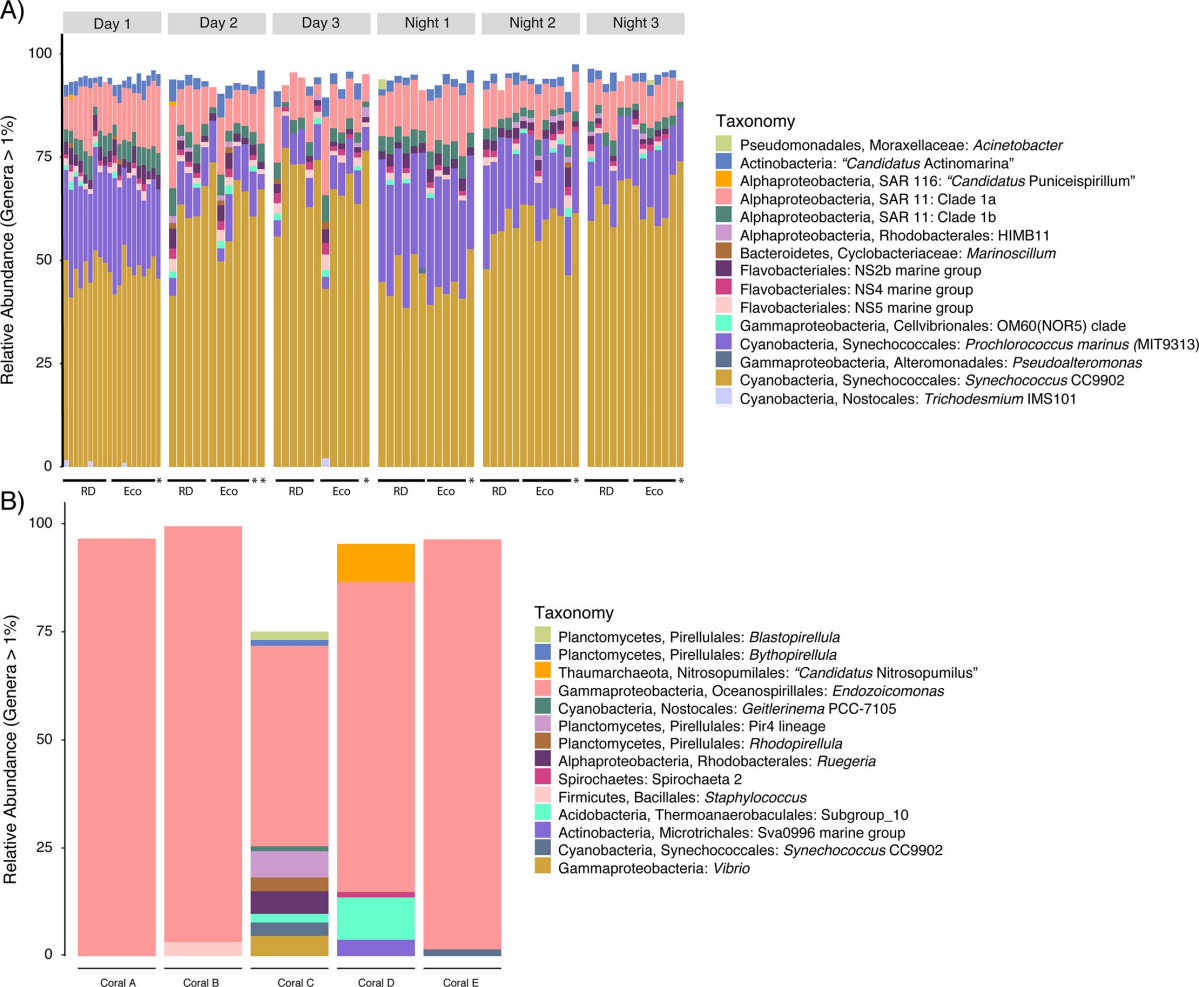
Relative abundances of bacterial and archaeal sequences from SSU rRNA gene sequencing that comprise >1% of the genus-type level community composition in A) reef-depth (RD), coral ecosphere (Eco) and surface seawater samples (*) and B) coral tissue samples. Colors indicate the taxonomic grouping at the genus-type level. Color legends are specific for each plot.

In terms of taxonomic composition, the average relative abundance of sequences identified as *Synechococcus* CC9902 was higher (30%) than *Prochlorococcus marinus* (MIT9313) (13%) across the time-series (Fig 7A). The average relative abundance of *Prochlorococcus marinus* (MIT9313) was higher at night compared to the day (1.2 times higher). Additionally, the relative abundance of *Synechococcus* CC9902 increased both at night relative to day (1.2 times higher) and over the entire study, aligning with the observed changes in cell abundances of these two groups (Fig 7A). Flavobacteriales and SAR11 sequences (average relative abundances of 5% and 12%, respectively) were detected across samples, with SAR11 clade 1a sequences being more abundant than clade 1b (Fig 7). Rhodobacterales HIMB11 sequences were absent during day 1 and night 1, but were detected during subsequent days and nights at low relative abundances (Fig 7). Sequences identifying as Cellvibrionales OM60 (NOR5) clade were also detected sporadically and at low relative abundances during all sampling time points. *Endozoicomonas* and *Vibrio* sequences were detected within a majority of the coral ecosphere and reef-depth samples at very low average relative abundances (0.11% and 0.09%, respectively). The coral tissue microbial communities were mostly dominated by *Endozoicomonas* (average relative abundance of 54%, Fig 7B). Colony C had a more diverse composition compared to the other colonies and *Vibrio* was detected at a low relative abundance of 0.5% in this colony (Fig 7B). No ASVs were shared between coral ecosphere or reef-depth seawater and coral tissue.

### Differential enrichment of taxa

**Fig 8:**
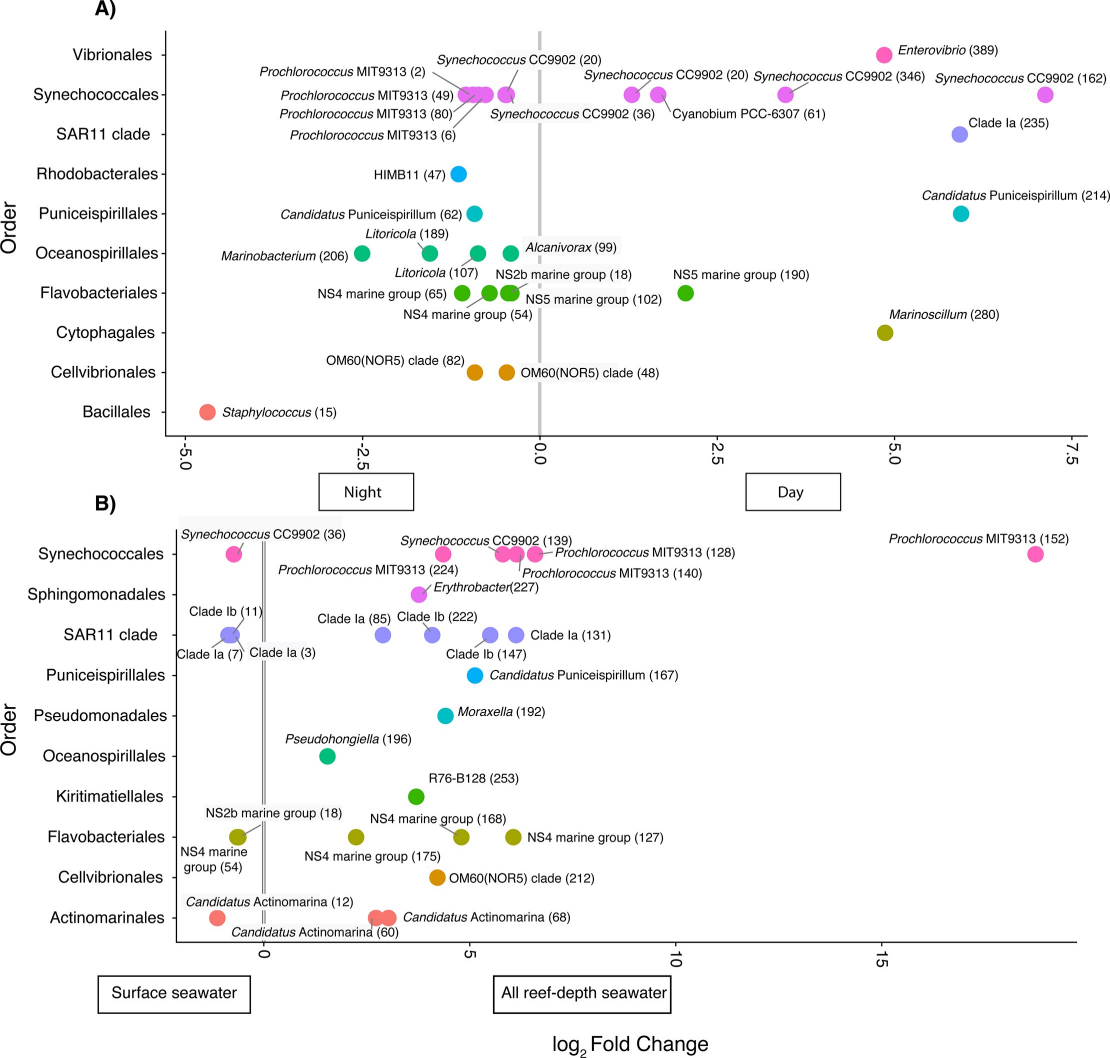
Significantly differential enrichment of bacterial and archaeal SSU rRNA gene amplicon sequence variants (ASVs) across A) reef-depth and coral ecosphere samples collected during the day or night and between B) all bottom reef seawater (reef-depth and ecosphere samples) and surface seawater. Each point represents an individual ASV labeled at the genus-type taxonomic level and the color reflects the order. Only ASVs matched at the genus level are plotted. The number in parentheses indicates the unique number assigned to each ASV sequence.

Differential enrichment tests revealed that there were 28 significantly enriched ASVs identified to the level of genus (p<0.05) between day (9) and night (19) reef-depth and coral ecosphere seawater bacterial and archaeal communities (Fig 8A, see S1 Appendix for ASV sequences). Interestingly, there were 3 *Synechococcus* CC9902 ASVs that were enriched during the day, whereas 3 *Synechococcus* CC9902 and 4 *Prochlorococcus marinus* (MIT9313) ASVs were enriched at night (Fig 8A). Gammaproteobacteria *Marinobacterium*, *Litoricola* (2 ASV sequences), and *Alcinovorax* within the order Oceanospirillales were enriched at night (Fig 8A). OM60 (NOR5) clade, *Staphylococcus*, NS4, NS5, and NS2b marine group, HIMB, and “*Candidatus* Puniceispirillum” ASVs were also enriched in samples collected at night (Fig 8A). In contrast, *Enterovibrio*, SAR11 clade 1a, and *Marinoscillum* ASVs were only enriched during the day and the fold changes were higher (Fig 8A).

Differential enrichment tests were also conducted between bottom reef seawater (including reef-depth and coral ecosphere communities) and surface reef seawater (Fig 8B). There were 20 significantly enriched ASVs identified at the level of genus in bottom reef seawater compared to surface reef seawater (7 enriched ASVs in surface reef seawater) (Fig 8B). Overall, ASVs identified as *Prochlorococcus marinus* (MIT9313), SAR 11 clades 1a and 1b, NS4 marine group, and “*Candidatus* Actinomarina” were more enriched in bottom reef seawater (Fig 8B). No ASVs were differentially enriched between coral ecosphere and reef-depth seawater microbial communities by both day and night or when tested individually by either day or night.

### Taxa exhibiting diel and daily rhythms

Ten ASVs exhibited significant rhythmicity in reef-depth and coral ecosphere seawater (Table 3). *Prochlorococcus marinus* (MIT9313) and *Synechococcus* ASVs accounted for a majority (80%) of the taxa that underwent significant synchronous changes in abundance over 24 hours across both seawater environments (Table 3, see S1 Appendix for ASV sequences). Interestingly, *Prochlorococcus marinus* (MIT9313) ASVs experienced changes in abundance over a phase of 24 hours compared to *Synechococcus* ASVs which experienced a phase of 12 hours. Aside from *Prochlorococcus marinus* (MIT9313) and *Synechococcus*, ASVs identified as *Cyanobium* PCC-6307 exhibited rhythmic patterns in reef-depth and coral ecosphere seawater. An ASV identifying within the Pirellulaceae family only experienced rhythmicity in reef-depth seawater (Table 3).

**Table 3:** Amplicon sequence variants (ASVs) that displayed rhythmic fluctuations in relative abundance over a period of 24 hours in reef-depth and coral ecosphere seawater.

Taxonomy	ASV #	Environment	Phase	p-value
*Prochlorococcus marinus* (MIT9313)	ASV2	reef-depth, ecosphere	24	2.62E-08, 2.49E-07
*Prochlorococcus marinus* (MIT9313)	ASV6	reef-depth, ecosphere	24	8.78E-07, 5.71E-05
*Synechococcus* CC9902	ASV43	reef-depth, ecosphere	12	9.17E-08, 1.14E-04
*Prochlorococcus marinus* (MIT9313)	ASV49	reef-depth, ecosphere	24	2.49E-07, 3.57E-06
Cyanobium PCC-6307	ASV61	reef-depth, ecosphere	12	8.78E-07, 1.27E-06
*Prochlorococcus marinus* (MIT9313)	ASV80	reef-depth	24	1.82E-06
*Prochlorococcus marinus* (MIT9313)	ASV128	reef-depth	24	2.70E-05
*Synechococcus* CC9902	ASV139	reef-depth	12	2.00E-03
Planctomycetacia, Pirellulaceae	ASV149	reef-depth	12	4.00E-04
*Synechococcus* CC9902	ASV159	reef-depth, ecosphere	12	8.97E-06, 5.87E-04

## Discussion

This study examined diel and daily variation in reef seawater microbial communities and inorganic nutrient concentrations and also investigated how these factors changed over three spatial scales: near-coral, reef-depth, and surface seawater. Overall, the marine picocyanobacteria *Prochlorococcus* and *Synechococcus* exhibited consistent fluctuations in cell abundance over diel and daily time-scales. Bacterial and archaeal alpha diversity was higher in reef-depth and coral ecosphere seawater compared to surface seawater and overall diversity decreased at night. Bacterial and archaeal community composition of coral ecosphere and reef seawater microbial communities were generally compositionally similar, but there were consistent changes in the relative abundances of picocyanobacteria and differential enrichment of select taxa between day and night (in reef-depth and ecosphere seawater), as well as between surface and reef-depth seawater. Lastly, there were several *Prochlorococcus* and *Synechococcus* ASVs in reef-depth and coral ecosphere seawater that exhibited significant rhythmicity over time.

### Diel and daily shifts in *Synechococcus* and *Prochlorococcus* abundances

*Synechococcus* and *Prochlorococcus* cell abundances underwent strong fluctuations over the course of the time-series and increased at night relative to day. Patterns of picocyanobacterial cell division and cell-cycling have been comprehensively documented in pelagic and oligotrophic regions of the Pacific and Atlantic [29, 62–64], but not in tropical and coastal coral reef ecosystems. Our observations of increasing abundances of *Synechococcus* and *Prochlorococcus* during the night can be explained by the synchronization between light irradiance, the cell cycle [65], and circadian rhythms in *Synechococcus* populations [66]. *Prochlorococcus* do not have true circadian rhythms, but do oscillate over a diel cycle with the influence of environmental cues [66]. Cell abundances for both populations were higher at night compared to their daytime abundances, but the populations were not monitored continuously between these times so peak cell abundances could not be estimated. Our data correspond with previously reported diel patterns of cell division in *Synechococcus* and *Prochlorococcus* populations measured in the eastern equatorial Pacific [62], but not with patterns of *Prochlorococcus* abundance in the North Pacific, where populations reached peak abundance during the day in warmer water [63]. The decrease in cyanobacterial abundance during the day could be attributed to physical movement of cells, grazing activities, and/or viral lysis [62], but we did not measure these factors here.

Over the course of three days, the abundance of *Prochlorococcus* decreased whereas the abundance of *Synechococcus* increased significantly and there were no correlations between picoplankton abundance and inorganic macronutrient concentrations when all time points were analyzed together. However, when samples were only compared by either day or night, *Prochlorococcus* abundance was correlated with temperature while *Synechococcus* abundance was correlated with nitrite and silicate concentrations at night, aligning with previous observations [63, 67, 68].

*Synechococcus* cell abundance was significantly lower in seawater collected surrounding colony E compared to three other colonies across the reef. This observation is interesting because it highlights differences between *Prochlorococcus* and *Synechococcus* distributions across the reef and in proximity to coral colonies and the reef substrate. This difference could potentially be attributed to selective grazing of these cells by nearby reef organisms and/or specific environmental conditions on this particular area of the reef. Nevertheless, this finding highlights the heterogeneity of the reef microbial community over space and the need to investigate the causal, ecological factors that impact this heterogeneity.

### Picoeukaryote abundances: Daily changes and differences by colony

Picoeukaryotic cell abundances increased steadily from day 1 to day 3, but did not change significantly between day and night. Additionally, the PCA revealed a correlation between picoeukaryotic cells and nitrate concentrations over the course of the study. This trend of increasing picoeukaryote abundances is unlikely to be a methodological artifact of sampling close to the reef benthos over the course of three days and nights because divers carefully sampled ecosphere and reef-depth seawater by maintaining neutral buoyancy and by minimizing contact with the benthos. Furthermore, if sediment disturbance was the reason for an increase in picoeukaryotes over time, a subsequent increase in unpigmented cells could also be expected, but this was not the case. As such, the change in picoeukaryote abundances over time is likely due to ecological variation rather than diver interference and disturbance.

Also of note are the differences observed in picoeukaryote cell abundances by coral colony over the course of the time-series. This observation is intriguing and opens up questions into how corals potentially influence surrounding phytoplankton cells and/or the distribution of picoeukaryote cells across the reef. Picoeukaryotic cell dynamics are generally underexplored in reef environments and our results indicate that these cells should also be included in ecological studies of coral reef microbial communities.

### Diel shifts in microbial community composition and diversity

Population fluctuations of *Synechococcus* and *Prochlorococcus* were also evident in the sequence data. Additionally, changes in cell abundances corresponded with changes in the relative abundance of these two groups, demonstrating coherence between flow cytometry and 16S rRNA gene amplicon sequencing in this case. Stemming from these patterns, decreases in observed bacterial and archaeal community diversity (richness and Shannon’s diversity) at night may partly reflect cell division dynamics in *Synechococcus* and *Prochlorococcus*. Logically, as *Synechococcus* and *Prochlorococcus* populations divide and increase in abundance, the overall diversity of the community decreases because the picocyanobacteria comprise more of that community. That being said, this trend did not change when we temporarily removed *Prochlorococcus* and *Synechococcus* ASVs from diversity analysis, demonstrating that overall changes in diversity occurred across the entire community and not just in the dominant picocyanobacterial populations.

Differential enrichment tests of reef seawater bacterial and archaeal communities (reef-depth and coral ecosphere) between day and night revealed ASVs exhibiting diel enrichment. Excluding the consistent diel changes in *Synechococcus* and *Prochlorococcus*, more ASVs within the orders Oceanospirillales, Flavobacteriales, Puniceispirillales, and Cellvibrionales were enriched at night. During the day, a few ASVs within the same orders of Puniceispirillales and Flavobacteriales were also enriched in addition to Vibrionales, the SAR 11 clade, and *Marinoscillum*. There are very few reports of differentially enriched taxa between day and night in reef seawater [e. g. 26, 30], but some of the trends we observed in this study have been observed in other marine microbial communities. Gammaproteobacteria exhibited higher activity at night [69] in the Mediterranean, corresponding with potential DOM release from grazing zooplankton. In our study, it is possible that enrichment of Oceanospirillales also indicates enhanced grazing on the reef at night. SAR11 clade bacteria were enriched during the day in reef seawater, aligning with decreases in SAR11 relative abundances at night in the English Channel [70] and up-regulated gene transcription in SAR11 during the day in the North Pacific Subtropical Gyre [71]. These trends could be explained by the dependence of SAR11 on sunlight for driving proteorhodopsin activity [72, 73].

### Heterogeneity in bacterial and archaeal diversity and composition across the reef

The observed richness of bacterial and archaeal ASVs was higher in seawater collected from reef-depth and coral ecosphere seawater relative to surface seawater during both day and night, demonstrating spatial heterogeneity in the microbial community over the water column and elevated microbial diversity at depth. Enhanced diversity closer to the reef may reflect a wider variety of microbial niches, increased nutrient availability, as well as less photoinhibition compared to surface seawater. Taxa enriched in reef-depth and coral ecosphere seawater compared to surface seawater bacterial and archaeal communities included *Prochlorococcus*, SAR11 (clades 1a and 1b), the NS4 marine group, and “*Candidatus* Actinomarina,” taxa that are associated with oligotrophic environments and that are commonly detected in reef seawater.

*Prochlorococcus* ASVs were identified as *Prochlorococcus marinus* (MIT9131), a low-light adapted *Prochlorococcus* ecotype. We suspect that there are more *Prochlorococcus* ecotypes in reef seawater, but did not have the resolution to capture this diversity by comparing differences in the V4 region of the 16S rRNA gene because ecotypes can differ by <1% variation [reviewed by 74]. Compared to other *Prochlorococcus* ecotypes, MIT9313 is usually found at the base of the euphotic zone and has the genetic capability of using and reducing nitrite as a source of nitrogen [75, 76]. *Prochlorococcus* could be advecting onto the reef from offshore currents, but if this were the case, we would expect *Prochlorococcus* to be evenly distributed across the shallow water column. We consistently detected this ecotype at 7 m depth as opposed to 100 m in the open ocean [reviewed within 77], demonstrating that reef-depth seawater has unique attributes that may select for the growth of this ecotype or that surface reef seawater is less hospitable to *Prochlorococcus* because of photoinhibition [78].

Like *Prochlorococcus*, heterotrophic bacteria within the SAR11 clade are abundant in oligotrophic marine environments and coral reef seawater [32, 79]. In fact, coral reef exudates from *P*. *astreoides* stimulate SAR11 growth rates and there is evidence that *P*. *astreoides* also grazes on these cells in mesocosm experiments [80]. Enrichment of SAR11 within reef-depth and coral ecosphere seawater suggests that substrates that accumulate within the reef benthic boundary layer could contribute to the presence or growth of SAR11 in reef seawater.

NS4 marine group bacteria within the phylum Bacteroidetes are commonly detected in marine microbial communities [81–83] and exhibit seasonality in some environments [84]. Bacteria within the “*Ca*. Actinomarina” are very small and their distribution tracks with abundances of *Synechococcus* [85]. “*Ca*. Actinomarina” have also been identified in coral reef seawater and coral mucus previously [45]. We have shown that both these groups are enriched in reef-depth seawater, expanding our knowledge about the biogeography of these taxa and their potential association with the reef.

Coral ecosphere and reef-depth seawater bacterial and archaeal communities had generally similar compositions and metrics of alpha diversity (richness and Shannon’s diversity index) even though distance (reef-depth or coral ecosphere) significantly influenced community similarity as revealed by the PERMANOVA test. Furthermore, no ASVs were significantly enriched in coral ecosphere compared to reef-depth seawater. These conflicting results potentially suggest that minor compositional differences do exist between ecosphere and reef-depth seawater, but that most of the compositional differences are driven by temporal changes. To elaborate, we expected to find taxonomic differences between these two seawater environments as well as enrichment of Gammaproteobacteria within the coral ecosphere seawater based on previous observations [32, 37], but did not observe these trends. This could be for several reasons including that *P*. *astreoides* does not influence coral ecosphere bacterial and archaeal communities to the same degree that other coral species do. For example, *P*. *astreoides*’ ecosphere bacterial and archaeal communities sampled in Cuba were more similar to reef-depth seawater microbial communities compared to ecosphere communities sampled surrounding other Caribbean coral species including *P*. *astreoides*, *Orbicella faveolata*, *Montastrea cavernosa*, *Pseudodiploria strigosa*, and *Acropora cervicornis* [37]. The second potential explanation is that associations between Gammaproteobacteria and *P*. *astreoides* could be site-specific and depend on local environmental conditions and/or on the health state of the coral colony, similar to observations that have been made about common coral-associated microorganisms [37, 86]. For instance, the coral colonies sampled within Cuba by Weber et al. [37] were located on more continuous reef structure with higher coral cover compared to the corals sampled in this study, which were located adjacent to sand patches. Differences in coral density (potentially influencing the source, supply, and detection of potential coral-associates or pathogens) or hydrodynamics could play a role in the differences reported here. It is interesting to note that the tissue microbiomes of *P*. *astreoides* were mostly dominated by *Endozoicomonas* bacteria whereas *Endozoicomonas* were present but cryptic (average relative abundance of 0.11%) in coral ecosphere and reef-depth seawater. These findings demonstrate that interactions between corals and planktonic microorganisms may be nuanced and depend on factors that have been previously unexplored, necessitating more research in this area.

## Conclusions

High-resolution sampling of surface, reef-depth, and coral ecosphere seawater microbial communities revealed several novel observations of seawater microbial dynamics on tropical coral reefs. Even though reef seawater bacterial and archaeal communities were highly similar over three days, there were consistent increases in *Prochlorococcus* and *Synechococcus* cell abundances at night and changes over time that corresponded with changes in temperature and increases in nitrite and silicate. We also identified diel patterns in bacterial and archaeal diversity, enrichment of different taxa by day and night, and specific taxa exhibiting rhythmic population fluctuations in reef-depth and coral ecosphere seawater. Bacterial and archaeal alpha diversity was higher in reef-depth seawater compared to surface seawater, suggesting that there may be enhanced microbial niches close to the reef, a hypothesis held widely, but rarely observed. Comparatively, temporal changes superseded spatial differences in terms of influence on the seawater microbial community, emphasizing the need for a more comprehensive understanding of how these communities change over short term (tidal cycle to days to weeks) and longer term (seasonal to annual) time-scales. Coral ecosphere and reef-depth seawater bacterial and archaeal communities had generally similar compositions in this study, suggesting that colony or site-specific conditions may influence the outcome of coral-microbial interactions within the coral ecosphere. Overall, these findings demonstrate the small-scale population dynamics that take place over a diel cycle and the relative influence of temporal compared to spatial changes on microbial communities sampled across one reef.

At the scale of the reef, understanding variability in microbial composition as well as dominant forcings on these communities is essential for describing baseline temporal and spatial dynamics in productive, diverse, and sensitive coral reef ecosystems. As coral reefs continue to decline, these changes may not only impact coral reef health and the composition of microbial communities on reefs, but also the variability of microbial population fluctuations.

## Supporting information

S1 Fig Comparison of ammonium (NH4+) concentrations between reef-depth and ecosphere seawater samples. Each point represents a sample. Point shape corresponds to sampling distance from the coral and point color reflects the colony adjacent to where sampling was conducted. Gray shading indicates samples collected at night. Lower and upper edges of the boxplot correspond to the first and third quartiles, the whiskers extend to the largest or smallest value at 1.5 times the interquartile, and the black bar across the box represents the median.
(DOCX)

S2 Fig Comparison of nitrite (NO_2^-^_) concentrations over three days. Each point represents a sample. Point shape corresponds to sampling distance from the coral and point color reflects the colony adjacent to where sampling was conducted. Gray shading indicates samples collected at night. Lower and upper edges of the boxplot correspond to the first and third quartiles, the whiskers extend to the largest or smallest value at 1.5 times the interquartile, and the black bar across the box represents the median.
(DOCX)

S1 Table Results of PERMANOVA (ADONIS) test examining factors influencing cell abundances, using 999 permutations. (DOCX)

S2 Table Results of PERMANOVA (ADONIS) test examining factors influencing macronutrient concentrations, using 999 permutations. (DOCX)

S1 Appendix (XLSX)
