# Supplementary material for: Diel, daily, and spatial variation of coral reef seawater microbial communities
Source: PLoS One. 2020 Mar 11;15(3):e0229442. doi: 10.1371/journal.pone.0229442 (PMC7065756; doi:10.1371/journal.pone.0229442)
Supplement: S1 Fig — Each point represents a sample. Point shape corresponds to sampling distance from the coral and point color reflects the colony adjacent to where sampling was conducted. Gray shading indicates samples collected at night. Lower and upper edges of the boxplot correspond to the first and third quartiles, the whiskers extend to the largest or smallest value at 1.5 times the interquartile, and the black bar across the box represents the median. (DOCX) [file pone.0229442.s001.docx]

Fig S1. Comparison of ammonium (NH4+) concentrations between reef-depth and ecosphere seawater samples. Each point represents a sample. Point shape corresponds to sampling distance from the coral and point color reflects the colony adjacent to where sampling was conducted. Gray shading indicates samples collected at night. Lower and upper edges of the boxplot correspond to the first and third quartiles, the whiskers extend to the largest or smallest value at 1.5 times the interquartile, and the black bar across the box represents the median.
