# Supplementary material for: Diel, daily, and spatial variation of coral reef seawater microbial communities
Source: PLoS One. 2020 Mar 11;15(3):e0229442. doi: 10.1371/journal.pone.0229442 (PMC7065756; doi:10.1371/journal.pone.0229442)
Supplement: S1 Table — (DOCX) [file pone.0229442.s003.docx]

| Factor | DF$ | Sums of Squares | MeanSqs§ | F model | R2 | Pr(>F)¶ |
| --- | --- | --- | --- | --- | --- | --- |
| Diel | 1 | 0.029 | 0.029 | 6.64 | 0.041 | 0.056 |
| Day | 2 | 0.050 | 0.025 | 5.74 | 0.072 | 0.043 |
| Distance^ | 1 | 0.019 | 0.019 | 4.22 | 0.026 | 0.171 |
| Colony‡ | 5 | 0.34 | 0.069 | 15.75 | 0.49 | 0.001 |
| Residuals | 59 | 0.26 | 0.0044 |  | 0.37 |  |
| Total | 68 | 0.71 |  |  | 1.00 |  |

Table S1. Results of PERMANOVA (ADONIS) test examining factors influencing cell abundances, using 999 permutations.
