# Supplementary material for: Diel, daily, and spatial variation of coral reef seawater microbial communities
Source: PLoS One. 2020 Mar 11;15(3):e0229442. doi: 10.1371/journal.pone.0229442 (PMC7065756; doi:10.1371/journal.pone.0229442)
Supplement: S2 Table — (DOCX) [file pone.0229442.s004.docx]

Table S2. Results of PERMANOVA (ADONIS) test examining factors influencing macronutrient concentrations, using 999 permutations.

| Factor | DF$ | Sums of Squares | MeanSqs§ | F model | R2 | Pr(>F)¶ |
| --- | --- | --- | --- | --- | --- | --- |
| Diel | 1 | 0.081 | 0.081 | 5.03 | 0.06 | 0.001 |
| Day | 2 | 0.17 | 0.086 | 5.30 | 0.13 | 0.001 |
| Distance^ | 1 | 0.026 | 0.026 | 1.63 | 0.020 | 0.127 |
| Colony‡ | 5 | 0.054 | 0.011 | 0.67 | 0.040 | 0.920 |
| Diel: day | 2 | 0.083 | 0.042 | 2.58 | 0.062 | 0.003 |
| Diel: distance | 1 | 0.016 | 0.016 | 1.012 | 0.012 | 0.430 |
| Day: distance | 2 | 0.041 | 0.010 | 1.26 | 0.03 | 0.25 |
| Diel: day: distance | 2 | 0.052 | 0.026 | 1.60 | 0.038 | 0.078 |
| Residuals | 51 | 0.82 | 0.016 |  | 0.61 |  |
| Total | 67 | 1.35 |  |  | 1.00 |  |
